# Primary hemiarthroplasty for the elderly patient with cognitive dysfunction and a displaced femoral neck fracture: a prospective, observational cohort study

**DOI:** 10.1007/s40520-020-01651-8

**Published:** 2020-07-23

**Authors:** Ghazi Chammout, Paula Kelly-Pettersson, Carl-Johan Hedbeck, Henrik Bodén, André Stark, Sebastian Mukka, Olof Sköldenberg

**Affiliations:** 1 Department of Clinical Sciences at Danderyd Hospital, Division of Orthopaedics, Karolinska Institutet, Stockholm, Sweden; 2 Department of Surgical and Perioperative Sciences, Umeå University, Umeå, Sweden

**Keywords:** Hip fracture, Femoral neck fracture, Cognitive dysfunction, Dementia, Patient-reported outcomes, Hip function, Health-related quality of life

## Abstract

**Background:**

At least one-third of hip fracture patients have some degree of impaired cognitive status, which may complicate their postoperative rehabilitation.

**Aim:**

We aimed to describe the outcome for elderly patients with cognitive dysfunction operated with hemiarthroplasty (HA) for a femoral neck fracture and to study the impact postoperative geriatric rehabilitation has on functional outcome up to 1 year after surgery.

**Methods:**

98 patients with a displaced femoral neck fracture with a mean age of 86 years were included and followed up to 1 year. The outcomes were hip-related complications and reoperations, the capacity to return to previous walking ability, health-related quality of life, hip function and mortality.

**Results:**

The prevalence of hip complications leading to a major reoperation was 6% and the 1-year mortality rate was 31%. The lack of geriatric rehabilitation was correlated with poorer outcomes overall and those who receive geriatric rehabilitation were less likely to be confined to a wheelchair or bedridden at the 1-year follow-up.

**Conclusions:**

Hemiarthroplasty is an acceptable option for elderly patients with a displaced femoral neck fracture and cognitive dysfunction. A lack of structured rehabilitation is associated with a significant deterioration in walking ability despite a well-functioning hip. However, the causality of this could be due to selection bias of healthier patients being sent to geriatric rehabilitation.

## Introduction

In lucid, elderly patients, with a displaced femoral neck fracture (FNF), primary hip arthroplasty provides better hip function and significantly fewer reoperations compared to internal fixation [1–3]. However, the optimal surgical procedure for FNF in elderly patients with cognitive dysfunction remain controversial [2, 4, 5].

The prevalence of cognitive impairment among hip-fracture patients has been reported to be as high as 55% [6, 7]. Patients with cognitive dysfunction have a high mortality rate as well as a high rate of both general and fracture-related complications [8–11]. This subgroup of patients is often excluded from participation in clinical trials, and therefore, there is little knowledge concerning the risk factors and prognosis in this group of patients [12]. In randomized controlled trials (RCTs) comparing surgical treatment options for patients with cognitive dysfunction, the inability for this patient group to give informed consent, and thereby the practical inclusion rate, have severely limited both the number of included patients and the external validity of these trials. Previous studies comparing hemiarthroplasty (HA) and internal fixation, [13] for patients with cognitive dysfunction have thus shown contradictory results [2, 4, 5] with high reoperation rates regardless of surgical treatment. There is also a lack of evidence regarding the effect of postoperative rehabilitation in patients with cognitive dysfunction.

In this study, we aimed to describe the outcome for elderly patients with cognitive dysfunction operated with HA for a displaced (garden 3 and 4) [14, 15] FNF and to study the impact postoperative rehabilitation has on functional outcome.

## Methods

### Study design and setting

This single-centre, prospective, observational cohort study was conducted between September 2009 and March 2016 at the Orthopaedic Department at Danderyd Hospital in Stockholm, Sweden. The study adhered to the STROBE (Strengthening The Reporting of Observational Studies in Epidemiology) guidelines [16].

### Ethical statement

The study was approved by the ethics committee of the Karolinska Intitutet and was performed in accordance with the ethical standards laid down in the 1964 Declaration of Helsinki. All persons gave their informed consent prior to their inclusion in the study. For Patients with severe cognitive dysfunction their relative gave informed consent as approved by the ethical committee.

### Study subjects and eligibility criteria

All patients with a displaced FNF who were admitted to the hospital during the inclusion period were screened for participation in the study. The inclusion criteria were patients ≥ 65 years old with an acute displaced FNF who were independent walkers, with or without walking aids, with cognitive dysfunction, defined as a known diagnosis of dementia and/or defined by a Short Portable Mental Status Questionnaire (SPMSQ) score of 0–7 indicating cognitive dysfunction. Exclusion criteria were pathological fractures, osteoarthritis, rheumatoid arthritis, previous hip disorders and patients deemed unsuitable for participation. Informed consent was given by the patients or relatives.

### Surgery and postoperative rehabilitation

The same surgical teams at our hospital were used for the surgeries and the patients were operated by orthopaedic general trauma surgeons. A direct lateral approach was used. A tapered, cemented stem with a unipolar head (CPT stem, Zimmer, Warsaw, Indiana, USA) was used. The patients were encouraged to immediate weight bearing and there were no restrictions applied for range of motion.

The patients were discharged for rehabilitation at a specialized geriatric ward or returned to their home or nursing home. The allocation to the type of rehabilitation was not formally randomized but was rather dependent on the present occupancy at the geriatric unit, i.e., if there currently were beds available. The rehabilitation in the geriatric ward was individually adapted to the degree of cognitive dysfunction with the goal of restoring walking ability before discharge. The usual stay at the geriatric ward was 10 days before discharge to their nursing home. For those patients who were discharged after surgery directly to their nursing home, the rehabilitation varied depending on the availability of physiotherapy and whether the staff had sufficient time to help the patient implement the training programme. All nursing homes did not have a defined rehabilitation programme for patients with displaced femoral neck fractures.

### Variables

#### Outcomes

Main outcomes were the prevalence of all hip-related complications and reoperations. Other outcomes included the ability to return to previous walking status, mortality, health-related quality of life (HRQoL), hip function, pain numeric rating scale (PNRS) and adverse events during the study period. The unique Swedish civic registration number for all patients was used to verify complications in the Swedish Hip Arthroplasty Register (SHAR) and mortality in the Swedish Death Register. HRQoL was assessed with a generic instrument, the health section of the EuroQol (European Quality of life) 5 dimensions (EQ-5D) score. Hip function and walking ability were evaluated with the modified Harris hip score (HHS) [17–20]. To assess the status of the activities of daily living, the ADL index according to Katz [21]  was used. Pain in the involved hip was measured using the PNRS. We recorded all general adverse events.

#### Exposure and confounders

The cohort was divided into two groups, those who were allocated to geriatric rehabilitation after surgery, and those who were not (control group). Allocation was performed on availability, i.e., a formal randomization was not done. The availability was determined by the number of beds currently available at the geriatric ward.

#### Follow-up and data collection

Data were collected at the time of study inclusion, at the end of hospital stay and by visits to research nurses at 3 and 12 months after surgery. If the patient was unable to attend a follow-up visit, the family, caregiver or patients themselves were interviewed by telephone. All study data were collected and managed in Research Electronic Data Capture (REDCap) at Karolinska Institutet.

### Statistical analysis

Fisher’s exact tests were used to analyse correlations between ordinal data, and Student’s *t* tests were used to analyse scale variables. A binary logistic regression was used for evaluating the risk of being unable to walk at the 1-year follow-up. The factors used in the model were the exposure variable (geriatric rehabilitation after surgery [yes/no]), age, sex and pre-fracture walking ability and living condition. The data are presented as mean differences and odds ratios (ORs) and the uncertainty estimation with 95% confidence intervals (CIs). A *p* value < 0.05 was considered statistically significant. Statistical analysis was performed using SPSS Statistics software version 22.0.

### Source of funding

The study was funded by grants from the regional agreement on medical training and clinical research (ALF) and from Sven Noréns foundation.

## Results

### Patient flow and descriptive data

**Fig. 1:**
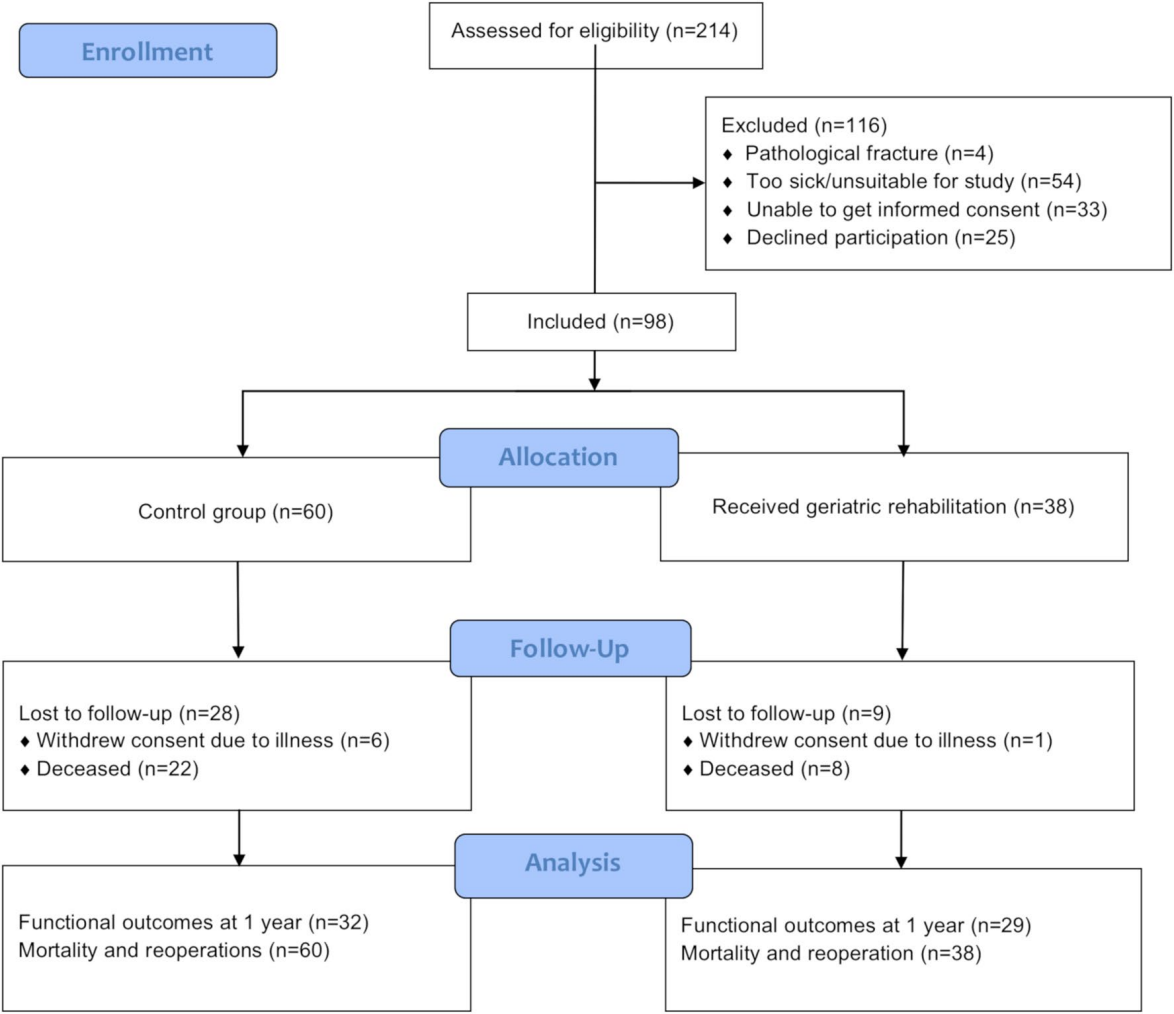
Flow of patients in the study

A total of 214 patients were screened during the inclusion period, and of these patients, 98 were recruited to participate in the study, 60 in the control group and 38 in the geriatric rehabilitation group (Fig. 1). The baseline demographics of the groups were similar but a higher proportion of the control group were admitted from nursing homes (Table 1). The overall mortality rate was 31% (30/98) with the control group having a slightly higher mortality rate at 1 years (36% versus 21%) but this difference did not reach statistical significance (*p* = 0.102, Fisher’s exact test).

**Table 1:** Baseline data

	Control group (*n* = 60)	Geriatric rehabilitation group (*n* = 38)	*p* value
Sex (*n*)			
Female	45	27	
Male	15	11	
Age (years)^b^	86 ± 6	85 ± 4	
Cognitive status (SPMSQ)			
Mild-moderate dysfunction (3–7)	6	4	
Severe cognitive dysfunction (0–2)	54	34	
ASA (*n*)			
1–2	12	12	
3–4	48	26	
BMI (kg/m^2^)^a^	23 ± 4	23 ± 4	
Functional class according to Charnley (*n*)^a^			
A	45	30	
B	10	8	
C	5	0	
Living conditions (*n*)^b^			< 0.001
Own home	7	29	
Nursing home^c^	53	9	
Radiographic classification			
Garden 3	10	6	
Garden 4	50	32	
Surgery time (min) ^a^	83 ± 25	79 ± 16	
Bleeding (mL)^a^	302 ± 135	334 ± 154	
Walking distance (*n*)			
Less than 0.5 km	38 (67%)	20 (53%)	
0.5–1 km	11 (19%)	11 (29%)	
1–2 km	6 (11%)	3 (8%)	
More than 2 km	2 (3%)	4 (10%)	

Numerical variables are presented as the mean and standard deviation and categorical variables are presented as numbers with percentages. *p* values ≤ 0.05 are presented

^a^Mean ± SD

^b^*p* value derived from Fisher’s exact test

^c^In Sweden nursing homes provides residential care for elderly patients who can no longer stay at their own homes. Nursing homes provide both assisted living and nursing care for those who do not need to be in a hospital, but cannot be cared for at home. All nursing homes have staff on hand 24/7

### Outcome data

#### Hip complications and reoperations

During the study period, eight patients (8%) suffered at least one hip-related complication, 5 (8%) in the control group and 3 (8%) in the geriatric rehabilitation group (Table 2). Two patients in the control group with recurrent dislocations were treated twice with closed reduction before revision surgery to a total hip arthroplasty with a dual-mobility cup.

**Table 2:** Hip-related complications during the study

Complications	Control group (*n* = 60)	Geriatric rehabilitation group (*n* = 38)
Reoperation (*n*)		
Dislocation	2	0
Periprosthetic fracture	2	1
Superficial infection	1	1
Deep infection	0	1
Total number of hip complication	5	3
Number of patients with any hip complication	5 (8%)	3 (8%)

Three periprosthetic fractures occurred in patients who had received a tapered CPT stem, two in the control group and one in the geriatric rehabilitation group. All were treated with open reduction and internal fixation. One patient in the geriatric rehabilitation group suffered a periprosthetic joint infection that healed after debridement and antibiotics for 3 months.

#### Walking ability

One year after surgery, 19% (19/61) of the surviving patients were either bedridden or wheelchair bound. The capacity to return to preoperative walking ability was diminished over the study duration; only 51% of the patients in both groups returned to their previous walking ability. The geriatric rehabilitation group had a statistically significant lower risk of being confined to a wheelchair or bedridden at the 1-year follow-up (odds ratio: 0.25; 95% CI 0.08–0.82; Table 3).

**Table 3:** Crude and adjusted logistic regressions for the risk of being either bedridden or wheelchair bound for the 61 patients that attended the 1-year follow-up

Variable	*n*	Event	Crude		Adjusted		*p* value
			Est	95% CI	Est	95% CI	
Geriatric rehab							
No	32	16 (50%)	Ref		Ref		
Yes	29	3 (10%)	0.26	0.08–0.79	0.25	0.08–0.82	0.009
Age			0.98	0.89–1.07	0.95	0.86–1.05	0.161
Sex							
Female	52	16 (31%)	Ref		Ref		
Male	9	3 (33%)	1.33	0.93–3.37	1.5	0.35–6.39	0.587
Pre-fracture living condition							
Own home	27	5 (19%)	Ref		Ref		
Nursing home	34	14 (41%)	3.08	0.94–10.1	0.65	0.10–4.19	0.653
Pre-fracture walking ability			0.84	0.67–1.05	0.83	0.65–1.05	0.212

The number of patients at risk and events at 1 year are presented for dichotomous variables. The models are adjusted for by whether the patients were admitted to geriatric rehabilitation after surgery, age, sex, and their pre-fracture living conditions and walking ability

#### Functional outcome

**Fig. 2:**
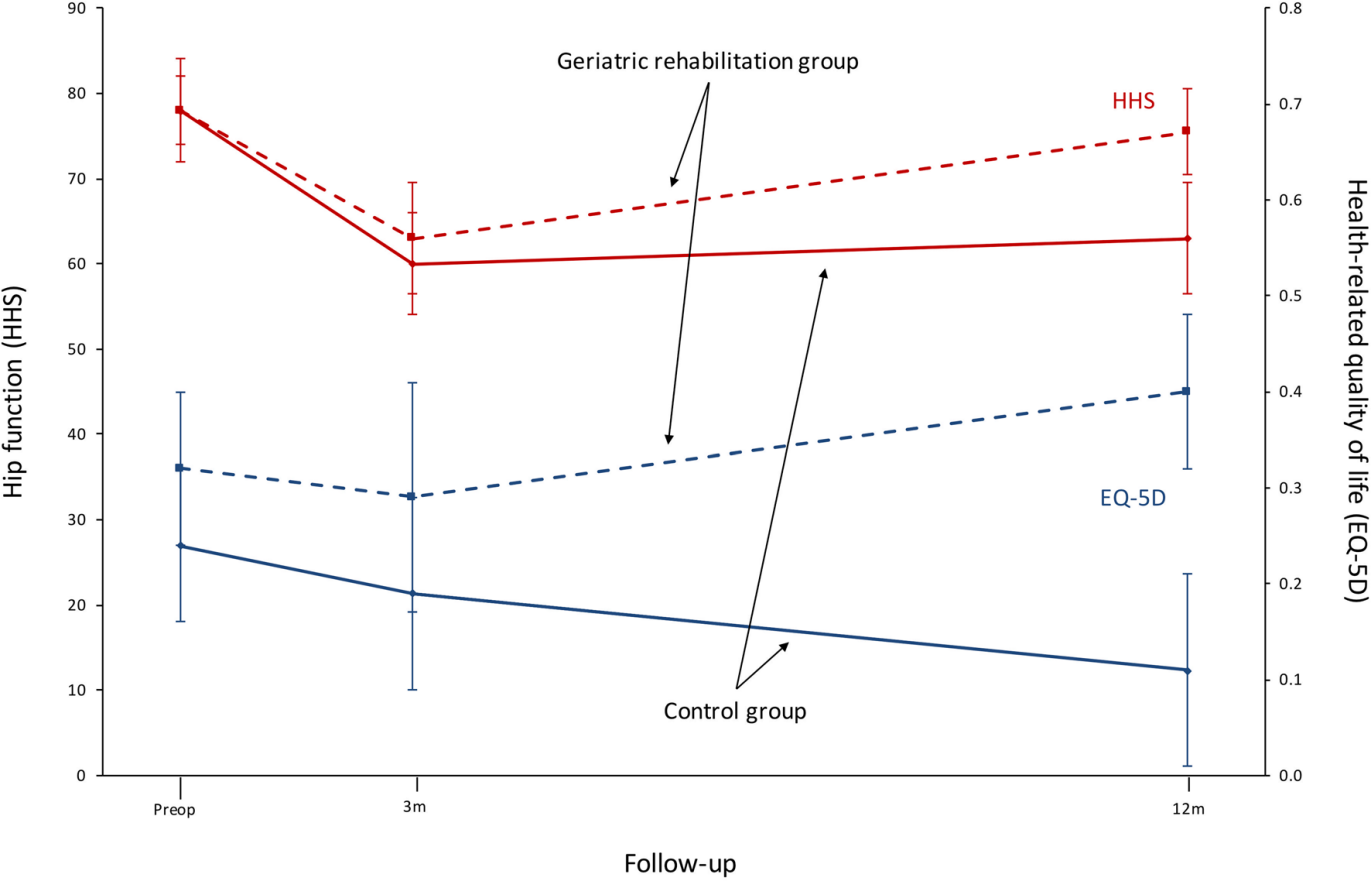
Line graph illustrating the mean values (and 95% confidence intervals) of health-related quality of life (HRQoL), hip function and pain scores during the study. Solid lines represent the control group and dotted lines represent the geriatric rehabilitation group. *EQ-5D* European Quality of life five dimensions, *HHS* Harris hip score

The functional outcome scores were similar in both groups at baseline (Table 4). The mean HRQoL declined from baseline in the control group but increased slightly in the geriatric rehabilitation group with a clinically and statistically significant difference between the groups at 1 year (mean difference 0.30, 95% CI 0.07–0.15) (Fig. 2). Hip function as measured with HHS declined in the control group and remained unchanged in the geriatric rehabilitation group compared to baseline at 1 year (mean difference 13, 95% CI 4–21(Fig. 2). The pain scores were relatively unchanged during the study period and we found no statistically significant difference between the groups at any of the follow-ups (Table 4). The initial radiological classification of the fracture (garden 3 or 4) did not affect outcome.

**Table 4:** Functional outcomes during the study period

	Control group (*n* = 60)		Geriatric rehabilitation group (*n* = 38)		Mean difference (95% CI)	*p* value
		*n*		*n*		
EQ-5D						
Baseline	0.24 ± 0.29	56	0.32 ± 0.27	38	0.07 (− 0.04 to 0.19)	0.22
At 3 months	0.19 ± 0.28	39	0.29 ± 0.34	32	0.10 (− 0.04 to 0.19)	0.16
At 12 months	0.11 ± 0.27	32	0.40 ± 0.32	29	0.30 (0.15 to 0.45)	< 0.001
HHS						
Baseline	78 ± 14	56	78 ± 18	38	0 (− 7 to 7)	0.97
At 3 months	60 ± 16	39	63 ± 18	32	3 (− 5 to 11)	0.49
At 12 months	63 ± 17	32	75 ± 13	29	13 (4 to 21)	0.03
PNRS						
Baseline	1.1 ± 1.9	56	1.6 ± 2.6	38	0.5 (− 0.5 to 1.5)	0.31
At 3 months	2.1 ± 1.9	39	2.2 ± 2.4	32	0.1 (− 1.0 to 1.2)	0.83
At 12 months	1.7 ± 1.8	32	0.9 ± 2.4	29	− 0.8 (− 1.9 to 0.3)	0.17

Variables are presented as the mean, standard deviation and 95% confidence interval. *p* values are derived from the Student’s *T* test

*EQ-5D* European Quality of life five dimensions, *HHS* Harris hip score, *PNRS* Pain numerical rating scale

## Discussion

In this prospective observational study of elderly patients with cognitive dysfunction treated with cemented HA for a displaced FNF, we found an 8% prevalence of hip-related complications up to 1 year after surgery. We also found that patients who received geriatric rehabilitation despite their cognitive dysfunction were less likely to be confined to a wheelchair or to be bedridden than those who did not. Geriatric rehabilitation was also correlated with a better hip function and health-related quality of life up to 1 year after surgery.

There is a persistent controversy regarding the use of HA in elderly patients with dementia or cognitive dysfunction [2, 4, 5, 22]. In the hip-fracture population, mortality is high [23], and patients with hip fractures and a SPMSQ score below seven points have a twofold increased mortality rate during the first 2 years after surgery compared to that of those with a hip fracture and a SPMSQ score of 7 or higher [7]. The 1-year mortality in our study was 31% and that is similar to that which was reported by others [2, 4, 22].

The prevalence of hip-related complications and major reoperations was doubled in comparison to the prevalence reported by Hedbeck et al. [5] and less than that reported by Blomfeldt et al. [2]. The differences could be explained by the limited number of patients included in Hedbeck's study and the use of an outdated implant in Blomfeldt’s study. In our study, the main reason for reoperation was the occurrence of periprosthetic fractures.

The mean HRQoL and hip function in our study was better at 1 year in the geriatric rehabilitation group compared to controls. The overall hip scores and EQ-5D scores are similar to those reported from other cohorts [22, 24–26].

In the present study, we found a high prevalence (19%) of being unable to walk which is in line with the findings by Mukka  et al. [22]. In addition, despite the fact that all participants in the study were walkers prior to surgery, only 51% of the patients were able to return to their previous walking ability. These results conform with the study by Rogmark et al. [6]. The high incidence of deterioration in walking ability among patients with cognitive dysfunction reflects the natural process of dementia and cognitive impairment and the difficulty for these patients to understand and follow rehabilitation regimes. Another explanation could also be the lack of rehabilitation resources for this patient group. Most patients with dementia or cognitive dysfunction live in nursing homes and are often discharged early from the hospital without receiving adequate rehabilitation.

The ultimate goal of rehabilitation is to return the patient to pre-fracture mobility and to avoid wheelchair dependence [27]. However, due to the inherent characteristics of the elderly hip-fracture population with often pre-existing comorbid conditions, rehabilitation can be difficult. The presence of cognitive impairment can alter their understanding of the need for rehabilitation, especially when it is painful [28]. Dementia can also increase the risk of falling by negatively impacting the individual’s ability to recognize and negotiate hazards, contributing to impaired body awareness and judgement [29].

Patients with dementia are at risk of being excluded from rehabilitation because of their perceived limited capacity for adherence and mobility. In contradiction to these assumptions, this study has shown that patients with cognitive dysfunction can benefit from participation in rehabilitation programmes and can regain their pre-fracture function after rehabilitation, which is in accordance with the findings from other studies [30–33].

### Strengths and limitations

One of the main strengths of our study is its uniqueness, as it focuses on the patient outcome in cognitively impaired patients who have a displaced femoral neck fracture. Another strength is the large number of patients included in the study compared to that in previous studies. Additionally, all patients were monitored according to a strict protocol with regular follow-ups and validated outcome scores.

The short follow-up time could be seen as a limitation, but in this patient group, the short-term mortality rate is high, and therefore, it is important to present the 1-year results. The use of a proxy, i.e., relatives or significant others, to report a patient’s condition is a limitation, but it is the only possible method that can be used, and the method is well documented [34, 35]. Our allocation to geriatric rehabilitation can have, although unintentionally, introduced a selection bias where healthier/more fit patients were selected to geriatric rehabilitation. Although this is not reflected in the two groups baseline data and that we have adjusted for this in our statistically model, we still advise caution when interpreting our results as these can be the results of selection bias. In addition, we had no data on the type of rehabilitation, if any, that the nursing homes providing.

## Conclusion

Cemented hemiarthroplasty with a direct lateral approach is a good option for elderly patients with a displaced (garden 3 and 4) femoral neck fracture and cognitive dysfunction. The lack of structured rehabilitation after surgery is associated with a significant deterioration in walking ability, regardless of a well-functioning prosthetic joint. However, the causality of this could be due to selection bias of healthier patients being sent to geriatric rehabilitation.
